# Analysis Choices Impact Movement Evaluation: A Multi-Aspect Inferential Method Applied to Kinematic Curves of Vertical Hops in Knee-Injured and Asymptomatic Persons

**DOI:** 10.3389/fbioe.2021.645014

**Published:** 2021-05-14

**Authors:** Johan Strandberg, Alessia Pini, Charlotte K. Häger, Lina Schelin

**Affiliations:** ^1^Department of Mathematics and Mathematical Statistics, Umeå University, Umeå, Sweden; ^2^Department of Statistical Sciences, Università Cattolica del Sacro Cuore, Milan, Italy; ^3^Physiotherapy, Department of Community Medicine and Rehabilitation, Umeå University, Umeå, Sweden; ^4^Department of Statistics, Umeå School of Business, Economics and Statistics, Umeå University, Umeå, Sweden

**Keywords:** functional data analysis, anterior cruciate ligament injury, movement control, knee rehabilitation, biomechanics, interval-wise testing, leg comparisons

## Abstract

Three-dimensional human motion analysis provides in-depth understanding in order to optimize sports performance or rehabilitation following disease or injury. Recent developments of statistical methods for functional data allow for novel ways to analyze often complex biomechanical data. Even so, for such methods as well as for traditional well-established statistical methods, the interpretations of the results may be influenced by analysis choices made prior to the analysis. We evaluated the consequences of three such choices when comparing one-leg vertical hop (OLVH) performance in individuals who had ruptured their anterior cruciate ligament (ACL), to that of asymptomatic controls, and also athletes. Kinematic data were analyzed using a statistical approach for functional data, targeting entire curve data. This was done not only for one joint at a time but also for multiple lower limb joints and movement planes simultaneously using a multi-aspect methodology, testing for group differences while also accounting for covariates. We present the results of when an individual representative curve out of three available was either: (1) a mean curve (*Mean*), (2) a curve from the highest hop (*Max*), or (3) a curve describing the variability (*Var*), as a representation of performance stability. We also evaluated choice of sample leg comparison; e.g., ACL-injured leg compared to either the dominant or non-dominant leg of asymptomatic groups. Finally, we explored potential outcome effects of different combinations of included joints. There were slightly more pronounced group differences when using *Mean* compared to *Max*, while the specifics of the observed differences depended on the outcome variable. For *Var* there were less significant group differences. Generally, there were more disparities throughout the hop movement when comparing the injured leg to the dominant leg of controls, resulting in e.g., group differences for trunk and ankle kinematics, for both *Mean* and *Max*. When the injured leg was instead compared to the non-dominant leg of controls, there were trunk, hip and knee joint differences. For a more stringent comparison, we suggest considering to compare the injured leg to the non-dominant leg. Finally, the multiple-joint analyses were coherent with the single-joint analyses. The direct effects of analysis choices can be explored interactively by the reader in the Supplementary Material. To summarize, the choices definitively have an impact on the interpretation of a hop test results commonly used in rehabilitation following knee injuries. We therefore strongly recommend well-documented methodological analysis choices with regards to comparisons and representative values of the measures of interests.

## Introduction

Three dimensional (3D) human motion capture commonly involves tracking positions of multiple markers placed on the body. It generates vast data of complex multi-joint coordination, often displayed as time series of single joint angles. Such movement curves are used for instance to interpret, and evaluate movement performance in sports or in rehabilitation after disease or injury, e.g., following rupture of the anterior cruciate ligament (ACL) – a very common sports injury. For analysis of movement curves, there has been a growing interest in using more recently progressed statistical methods within the functional data analysis (FDA) framework. In the context of knee control and ACL injury evaluation, FDA-methods have been applied for analysis of movement curves from different hops tests like the vertical hop (Ryan et al., 2006), the one-leg hop for distance (Hébert-Losier et al., 2015), the drop jump (Hébert-Losier et al., 2018) and a novel standardized side hop (Markström et al., 2019).

When evaluating knee function after ACL injury, movement performance of individuals is usually tested in several different types of functional assessments, most commonly using hop tests (Abrams et al., 2014). Specifically, single-leg hop tests are interesting to study since they are more challenging from an (re-)injury risk perspective than for example walking, and also mimic sport participation to a higher degree (Rudolph et al., 2000). Here, we focused on the one-leg vertical hop (OLVH), a common single-leg hop test in clinics and research (Gustavsson et al., 2006; Markström et al., 2021b). Each single functional assessment, observed using a motion capture system, results in multiple movement curves corresponding to different joints, different movement planes or different phases of the task.

In the present paper, we compared the hop performance of individuals in three groups: those who had ACL reconstruction (ACLR), and two control groups (athletes and non-athletes). Control group comparisons are of interest for many reasons. Firstly, comparing performance of the injured to the non-injured leg within an individual, for example using the commonly applied measure of limb symmetry index, has been proven unsatisfactory due to bilateral deficits or lack of consensus of relevant specific outcome measures (Gokeler et al., 2017; Wellsandt et al., 2017). Secondly, the comparison with elite athletes is usually missing in knee rehabilitation research despite its importance in assessing suitability for return to elite sports.

Prior to any comparisons, several methodological decisions must be made, for example how to best quantify data, how to choose representative measures, or how to make comparisons, if that is part of the research questions. Such analysis choices may have substantial influence on the final results and thereby the conclusions. A recent paper in Nature demonstrated that when 70 research groups were given a common brain imaging data set, no teams chose identical workflows to analyze the data and as a consequence the results varied (Botvinik-Nezer et al., 2020). In the field of biomechanics, there is surprisingly little scientific discussion on the influence and consequences of methodological choices, both for analyses of the traditional discrete event-related kinematic variables (e.g., peak angles), commonly used for evaluating knee function after ACLR (Kotsifaki et al., 2020) and for analysis and interpretation of curve data comparisons.

For movement analysis, individuals are usually represented by either one single movement curve or by extracted variables derived from the time-series data (e.g., Ryan et al., 2006; Thomeé et al., 2012). Alternatively, an average curve or averaged event-related variables are used to represent individuals (e.g., Wang et al., 2013; Tate et al., 2017; Markström et al., 2019). To evaluate how consistently a movement is performed by an individual, analysis commonly involves either movement curves or event-related variables from repeated trials, representing movement variability (e.g., Fleisig et al., 2009; Preatoni et al., 2013). In either case, the process requires choices regarding the compilation of the specific outcome measures used in the analyses. With a focus on curve data analysis, we explored several important methodological choices of relevance to the workflow and results, all applied to our set of motion data. The first methodological question targeted in this paper was: *How does the choice of curve outcome measures influence the results?*

In research related to ACL injury, there are different ways of comparing injured groups to asymptomatic control groups. Some studies compare the injured leg to the non-dominant leg of controls (e.g., Markström et al., 2021a), some to the dominant leg (e.g., Hoffman et al., 1999; Setuain et al., 2019), while some reports match for leg dominance (Lehmann et al., 2017), and others use an average of both legs (Wang et al., 2013). The rationale for comparing the injured leg to the non-dominant leg is usually that it may be a more conservative choice (assuming a better motor control of the dominant leg), avoiding conclusions that are not true (i.e., type I errors). The dominant limb does not, however, always demonstrate superior control as for significant kinematic differences between the dominant and the non-dominant leg of asymptomatic individuals/athletes (e.g., Van der Harst et al., 2007; Greska et al., 2017; Mokhtarzadeh et al., 2017). Our second methodological question targeted in this paper was therefore: *How does the choice of leg comparison influence the results?*

To obtain a comprehensive understanding of the movement performance of an individual, it may be desirable to include a whole test battery. Still, analyses are often restricted to a single hop test. Even so, it is complicated to analyze movements of several joints of the body simultaneously. Here, we extend and apply a newly suggested multi-aspect methodology to analyze curve data from multiple joints and movement planes simultaneously (Pini et al., 2019). In short, we can then make statistical comparisons between groups, where individuals are represented by multivariate functional data, while accounting for covariates in the statistical model. The third, and last, methodological question targeted in this paper was: *How does the choice of different combinations of joints influence the results?*

The main aim of the current cross-sectional study was to explore how the results would vary depending on the choices of different alternatives for comparisons and representative values for measures of interest (outlined above). The results refer to the evaluation of the movement performance of the one leg vertical hop in a group who had had ACLR compared to athletic and non-athletic control groups with presumably a different level of knee performance. Commonly, in analyzing vertical hop performance across groups, one would choose to represent individuals using an average curve, and compare the injured leg for ACLR to the non-dominant leg for asymptomatic persons, and to perform analysis on movements of the trunk and the hip, knee and ankle joint, separately. The main reason here for comparing to the non-dominant leg is to have an analysis coherent with previous studies on the same cohort. Based on previous research, we anticipated that the ACLR group would have a distinguished movement pattern compared to controls and athletes in terms of increased trunk, hip and knee flexion and that athletes would jump higher and show less kinematic variability than the other groups. We thus illustrate and compare the results in the light of the different analysis choices. The analyses were performed on curve data from the highest hop (outcome measure *Max*), and the mean curve (*Mean*), both addressing the performance outcome. We also analyzed the variability curve (*Var*), representing the stability of the kinematic performance. Further, both the injured and the non-injured leg were compared to both the dominant and the non-dominant leg. All possible combinations of movements in one, two, three and four observed joints/segments (trunk, hip, knee, and ankle) were also analyzed.

A main component of the current paper is a feature illustrating the consequences of these research process choices on the outcomes. The reader can visualize all the results of the different analysis options, and/or select part of the results, using the so-called Shiny App provided on GitHub as Supplementary Material^1^.

## Materials and Methods

### Participants

In this study, ACLR are compared to asymptomatic controls (CTRL, not so physically active), and elite athletes (ATH, highly active, mainly in floor ball and soccer, in minimum the 2nd highest league, and assumed to have a superior movement control). In total, 76 participants (31 ACLR, 24 CTRL, and 21 ATH) performed the OLVH (Table 1). The only significant differences between the groups were found for the background variable BMI (where ACLR differed from CTRL). A previous power analysis for the ethical approval showed the need for 22 participants per group. The aim for the data collection was 30 to allow for drop out. We adhered to this power analysis and considered that the number of participants were sufficient for the current study. Prior to testing, all participants were interviewed and screened for exclusion criteria, and also underwent a clinical knee examination by an experienced physiotherapist. Exclusion criteria were any musculoskeletal or neurological pathology possibly affecting outcomes. The ACLR participants had suffered a unilateral ACL injury and had undergone reconstructive surgery (hamstring graft) on average 30.2 months prior to testing. They had also returned to previous activity levels. Leg dominance was determined as the preferred leg for kicking a ball. For the ACLR group, eight individuals had injured their non-dominant leg.

**TABLE 1:** Background characteristics for the individuals included in the statistical analysis (for at least one leg).

	**ACLR**	**CTRL**	**ATH**
Male/female	7/24	5/19	3/18
Right/left dominance	29/2	23/1	19/2
Age (y)	24.0 (4.6)	23.2 (3.27)	21.4 (2.87)
Height (cm)	172 (7.28)	170 (6.04)	172 (7.42)
BMI*	23.7 (2.34)	22.3 (2.02)	22.4 (1.94)
Max hop height (m) I/ND	0.23 (0.04)	0.23 (0.03)	0.23 (0.04)
Max hop height (m) NI/D	0.23 (0.03)	0.24 (0.05)	0.24 (0.03)

Data presented as number of individuals or mean (standard deviation). D: dominant leg, I: injured leg, ND: non-dominant leg, NI: non-injured leg. *Significant variable with respect to ANOVA, at α-level 0.05.

The study was approved by the Regional Ethical Review Board in Umeå, Sweden (Dnr. 2015/67-31). All participants provided prior written informed consent in agreement with the Declaration of Helsinki.

### Test Procedure

All participants performed the OLVH at the U-motion laboratory, Umeå, Sweden. The test protocol has been previously described in detail (see Markström et al., 2021b). Briefly, participants stood upright and barefoot on the testing leg with both hands behind the back holding a short rope (25 cm) in order to standardize the jump and not to occlude reflective markers on the body with the arms. The instructions were to jump upward as high as possible, land on the same leg with balance and to not release the rope, which also determined a successful jump. The participants had one or two practice trials for familiarization. Participants then performed three to five trials on each leg.

Movements were registered at 240 Hz using a motion capture system with eight cameras (Oqus 300, Qualisys AB, Gothenburg, Sweden) emitting infrared light. The details of the data collection and marker set, which included 56 passive reflective light spherical markers, are described in detail by Markström et al. (2021a). The same test leader applied markers and instructed all participants.

### Data Preprocessing

The software Qualisys Track Manager (v.2.2, Qualisys AB, Gothenborg, Sweden) and Visual3D (v.5.02.19, C-Motion Inc., Germantown, MD, United States) were used for data preprocessing. Angle data were filtered with a fourth-order bidirectional low-pass Butterworth digital filter with a cutoff frequency of 15 Hz. After data registration and preprocessing, data from 2 to 4 trials for each leg of each participant were deemed suitable for analyses. In the statistical analysis, participants were required to have three suitable trials. It is crucial to obtain the average, maximum, or variance of the same number of replicates for each individual. The reason for this is that the distribution of e.g., a mean, maximum or variance of three data points compared to that of two data points is not the same, which often is an underlying assumption of the statistical analysis. Hence, participants with only two observed replicates on a specific leg were removed from all analyses involving that leg. For participants with more than three replicates, only the first three were selected for the analysis. In summary, for the ACLR group, 27 and 25 participants were included for the injured and non-injured leg, respectively. For the ATH and CTRL groups, 19 and 22 participants were included for the dominant leg, while 17 and 19 were included for the non-dominant leg, respectively. For a few individuals, there were some joint curves with missing fragments due to hidden markers, but these were believed to be of minor importance for the data analysis.

**FIGURE 1:**
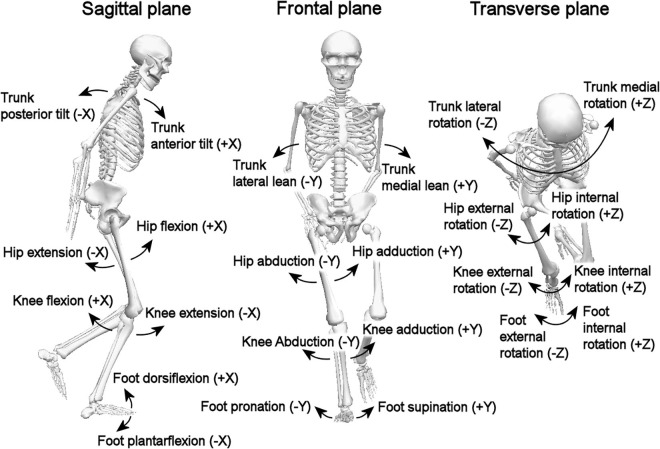
The segments/joints and planes.

In our analyses, we considered movements of the trunk, hip, knee and ankle in the sagittal, frontal, and transverse planes, for details see Figure 1.

**FIGURE 2:**
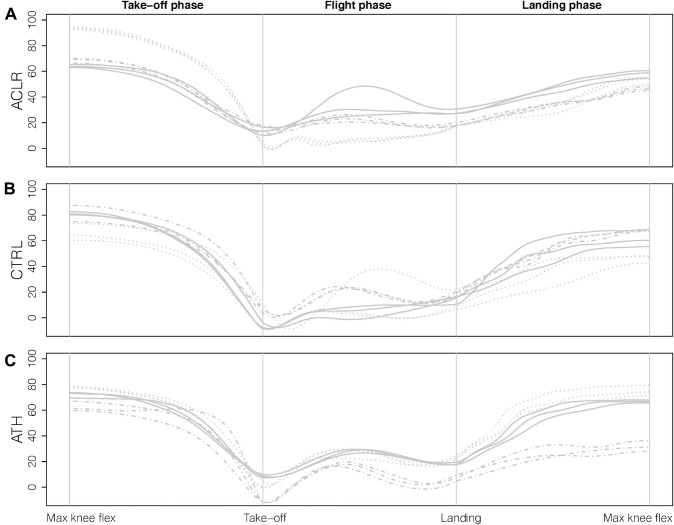
Illustration of the three phases and the landmarks (gray vertical lines) using knee angle data for the sagittal plane. For each group **(A)** ACLR, **(B)** CTRL, and **(C)** ATH, three repetitions for three randomly selected participants are displayed, where different line types correspond to different individuals.

The OLVH was divided into three phases: take-off (maximum knee flexion prior to take-off to take-off where vertical force < 20 N), flight (take-off to landing where vertical force > 20 N) and landing (landing to maximum knee flexion after landing), see Figure 2. Landmark registration was applied, implying that the start and the end of each phase occurred at the same relative time point for all participants. For statistical analysis, the domain was discretized with equally as many (101) time points in each phase.

### Statistical Analysis

In the following, we describe the multivariate FDA method that we applied to test for differences between ACLR, CTRL and ATH taking into account covariates (BMI, sex, and hop height). The same methodology was applied to the three types of representative curves of *Max*, *Mean*, and *Var* for each joint/segment and plane. For each participant, the multivariate functional data corresponds to several joints and planes (sagittal, frontal, and transverse), in this section referred to as *aspects*. The proposed methodology could be used also for *post hoc* analyses, by performing pairwise comparisons of groups with additional adjustments (e.g., using Bonferroni correction). Here, the main focus is on the overall group effect.

The method used here is an extension to functional linear models of the multi-aspect interval wise testing (IWT) procedure, described in Pini et al. (2019). The method allows for group comparisons of multivariate functional data accounting for covariates. In addition, it also enables identification of parts of the domain with significant differences. In the following paragraph, we briefly describe the extended multi-aspect IWT. The technical details of the procedure are presented as Supplementary Material.

The multi-aspect IWT is a method developed for testing simultaneously multiple aspects of functional data and adjusting for multiplicity. The idea behind the procedure is to provide adjusted *p*-value functions for testing statistical hypotheses over the domain. The methodology results in three different types of adjusted *p*-value functions.

• One overall multivariate adjusted *p*-value function, that is testing whether there is a difference between at least two groups, on at least one aspect included in the analysis.
• A collection of level-1 adjusted *p*-value functions, one corresponding to each aspect included in the analysis (further information below).
• A collection of level-2 adjusted *p*-value functions, one corresponding to each aspect included in the analysis (further information below).

All of the above *p*-value functions are adjusted using IWT (Pini and Vantini, 2017), to take into account the intrinsic multiplicity of functional data: data are theoretically infinite-dimensional objects observed along the domain. Pointwise (unadjusted) *p*-values cannot be used for domain selection, since they would not control the probability of finding false discoveries along the whole domain. Hence, a multiplicity adjustment is made so that all adjusted *p*-value functions are provided with a control of the interval-wise error rate; for an interval of the domain with no differences between groups, the probability that such an interval (or part of it) is selected as significant is controlled. In practice, significant parts of the domain are selected by applying a threshold (e.g., 5%) to the adjusted *p*-value function. The adjustment guarantees that the probability of erroneously selecting an interval where the groups do not differ is below 5%.

The level-1 adjusted *p*-value functions adjust solely for the domain. Level-2 adjusted *p*-value functions are also adjusted for the multiplicity that is due to the fact that several aspects of functional data are tested at the same time. This makes them more conservative than level-1 adjusted *p*-value functions, but the provided control is also stronger. For an interval of the domain with no differences between groups on any subset of aspects (e.g., no differences in the groups for the ankle in any of the planes), the probability that such an interval (or part of it) is selected as significant in at least one aspect is also controlled (it is below the significance level, e.g., 5%). Multivariate *p*-value functions adjust only for the domain, in a multivariate setting: if in an interval there are no differences between groups on all considered aspects (e.g., no group differences for the ankle in all planes), the probability that such an interval (or part of it) is identified as significant is controlled (it is below the significance level, e.g., 5%).

Functional data of each tested aspect were modeled as a linear combination of functional group effects plus scalar covariates’ effects multiplied by functional coefficients, as in Abramowicz et al. (2018). Then, group differences were tested using a pointwise test statistic that is based on the sum of squared differences between groups’ effects divided by the standard error of such differences (square of *t*-test statistics for group difference). So, the tests that we performed account for the presence of covariates, even though we decided not to test directly for any effects for covariates. Note that this would also be possible using the same methodology, but it is not within the scope of the present paper.

When performing the multivariate tests that are needed for the computation of the overall multivariate *p*-value function and for the level-2 adjusted *p*-value functions, the pointwise test statistics corresponding to single aspects were summed-up. Since all test statistics were standardized (i.e., the differences between groups’effects were divided by the corresponding standard error), the difference in scale between the aspects that we combined does not affect the results.

All statistical analyses were performed using R (version 3.6.1, R Core Team, 2019).

## Results

In traditional analysis, as described in the introduction, individuals would usually be represented by their average curve, and comparison of the injured leg for ACLR would be made to the non-dominant leg for asymptomatic persons, and the analysis would be carried out separately on selected joints/segments (trunk, hip, knee, and ankle). The summarized results for this choice scenario are highlighted in Table 2 by gridded cells (with red numbers). Significant group differences were identified for all joints, except the ankle, on at least some part of the curve domain. The main aim of this paper was, however, to illustrate how these results may change when we modify our analysis choices.

**TABLE 2:** The total percentages of the domain (corresponding to one or several parts of the domain) with significant differences (multivariate *p*-values < 0.05) between at least two of the three groups on at least one of the joint(s) and plane(s).

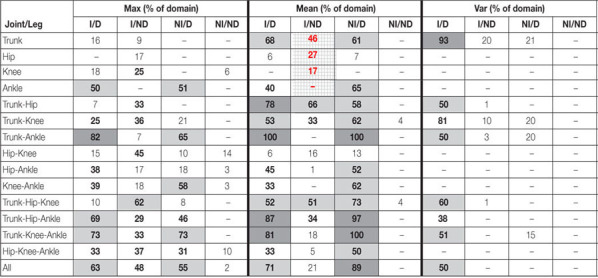

*Bold numbers indicate a total percentage over 25%, light gray cell indicates a total percentage over 50%, while dark gray indicates a total percentage over 75%. Gridded cells (with red numbers) correspond to the results of the traditional methodological choices (i.e., representing individuals with an average curve, compare the injured leg for ACLR to the non-dominant leg for asymptomatic persons for each joint separately). D: dominant leg; I: injured leg; Max: Curve corresponding to highest jump; Mean: Average curve from three trials; ND: non-dominant leg; NI: non-injured leg; Var: Variance curve based on three trials.*

A more complete summary of the results for all combinations of our different analysis choices is presented in Table 2, where each row corresponds to the analysis of the specified combination of joints. The three planes (sagittal, frontal and transverse) are per default included in all analyses. For each combination of joint movements, the table shows the percentage of the domain where the statistical test has detected a significant difference between the three groups (ACLR, CTRL, and ATH) for at least one joint and at least one plane. Such results are obtained by applying a threshold of 5% to the multivariate *p*-value functions corresponding to each combination of joints. The purpose of the table is to provide an overview whether, and if so how, the results change depending on the initial choices. The table only provides a rough summary and does not indicate any details of specific differences. The complete results are presented as interactive Supplementary Material (see text footnote 1), where the reader may visualize the results with more details. The reader can specify combinations of joints, outcome type, and which legs to compare and see results for all three types of adjustments.

### How Does the Choice of Curve Outcome Measure Influence the Results?

**FIGURE 3:**
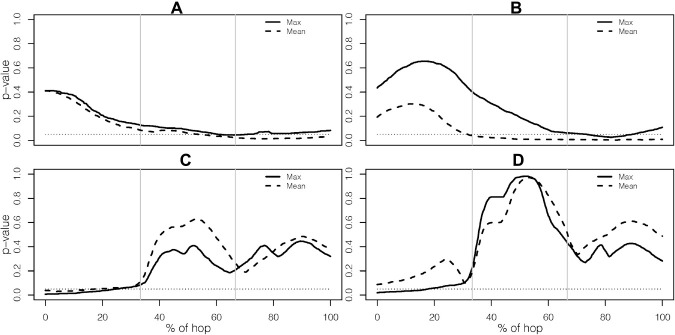
Multivariate *p*-value functions for outcome measure Mean and Max for **(A)** the trunk when comparing the injured leg to the non-dominant leg, **(B)** the trunk when comparing the injured leg to the dominant leg, **(C)** the knee when comparing the injured leg to the non-dominant leg, and **(D)** the knee when comparing the injured leg to the dominant leg. The dotted line indicates the level of 5%.

We found group differences when the analyses were based on the two outcome measures *Max* and *Mean*, with more pronounced differences when using the *Mean*, see Table 2. A thorough investigation of the complete results showed that the *p*-value functions, in many of the comparisons, had a similar shape. They were in many cases also close to the cut-off of 5% for parts of the domain. For instance, multivariate *p*-value functions for the trunk and knee are displayed in Figure 3. The *p*-value functions for the two outcome measures *Mean* and *Max* were similar (in shape), and in case of the trunk the signal was stronger (constantly lower *p*-value function) for the outcome measure *Mean*. With the outcome measure *Var*, representing the variability of the performance, we found fewer significant group differences.

### How Does the Choice of Leg Comparison Influence the Results?

For the ankle and the trunk separately, a larger total part of the domain showed significant differences when the injured leg was compared to the dominant leg instead of the non-dominant leg. For the knee and hip, the opposite was seen. A thorough investigation of the complete results (see text footnote 1) showed that for the hip, when comparing the injured leg to the non-dominant or to the dominant leg, different parts of the domain corresponding to significant group differences were identified. When comparing the non-injured leg to the dominant leg, there were differences in both the movements of the trunk and of the ankle, while the comparison of the non-injured leg to the non-dominant leg resulted in very few or no significant results depending on choice of outcome measure, see Table 2.

### How Does the Choice of Combination of Joints Influence the Results?

The analyses which used different numbers of joints are coherent in the sense that the results of the 1-joint analyses also influence the results of 2-, 3-, and 4-joint analyses. For example, if a joint is significant in the 1-joint analysis it is likely that it is also significant whenever it is included in the multivariate tests of two or more numbers of joints, and thus, strengthening the results of the findings for that particular joint.

### Specific Results for the Knee Joint

**FIGURE 4:**
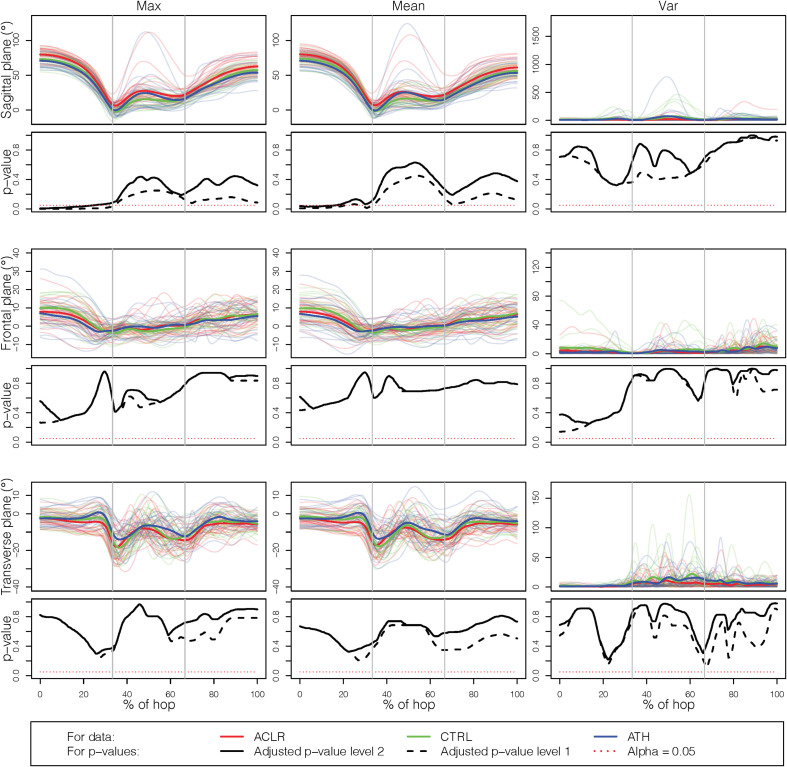
Results for the single knee joint, comparing the injured leg with the non-dominant leg. The rows correspond to the three planes (sagittal, frontal, transverse) while the columns correspond to the different outcome measures (Max, Mean, and Var).

**FIGURE 5:**
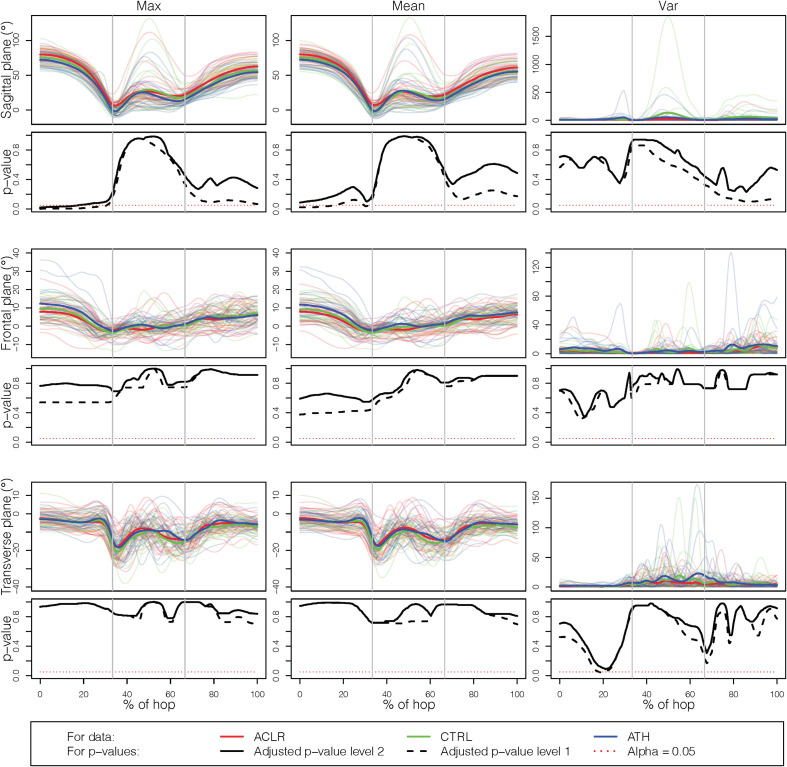
Results for the single knee joint, comparing the injured leg with the dominant leg. The rows correspond to the three planes (sagittal, frontal, and transverse) while the columns correspond to the different outcome measures (Max, Mean, and Var).

The results for the analysis based solely on the knee joint, still using all three planes, are presented with more details in Figures 4, 5. Figure 4 shows results for the comparisons of the injured leg of ACLR with the non-dominant leg of CTRL and ATH, while Figure 5 shows the corresponding results when the injured leg is compared to the dominant leg. The results in Figure 4 are very similar to those in Figure 5, and consistent with the results discussed in section “How Does the Choice of Curve Outcome Measure Influence the Results?”, the level-1 and level-2 adjusted *p*-value functions for the outcome measures *Max* and *Mean* have a similar shape, while the *p*-value functions for *Var* were different. In both comparisons, the group difference on outcome measures *Max* and *Mean* is mainly due to group differences in the sagittal plane at take-off, while there are no significant differences using *Var*. Finally, note that–with the only exception of the comparison between the injured and dominant leg with *Mean*–the level-2 adjusted *p*-value function is able to identify significant group differences at the 5% level even though it is more conservative than the level-1 adjusted *p*-value function.

## Discussion

The overall purpose of this paper was to evaluate the movement performance of the OLVH across three groups; ACL-injured individuals compared to athletic and non-athletic controls, respectively, with the main purpose of illustrating if, and how these results may change when the analysis choices are modified. There is a need for such a debate when interpreting movement performance in different populations, while the papers seem rather few. A recent study nevertheless addressed methodological choices in muscle synergy analysis and concluded that their results were sensitive to their choices and also highlighted the need for disclosure of all methodological aspects in studies (Banks et al., 2017). Our study indeed confirmed that the results and subsequent interpretations may vary as an effect of the methodological choices.

In line with the main aim and to keep the study report concise, we have intentionally not elaborated on all the specific findings for each of the analysis choices and their respective combinations, including on the potential resulting clinical implications. Although both of interest and importance, such discussion would need to be more extensive, and we considered it out of scope of the present study. However, we provide the possibility for the interested reader to fully investigate the specific results in the provided app in the Supplementary Material (see text footnote 1).

### Choice of Outcome Measures

The participants were rather consistent in how they performed the OLVH across repetitions, implying that the mean curve for each participant was similar to the curve corresponding to the highest jump (cf. Figure 2). The similar results for *Mean* and *Max*, when investigating the *p*-value functions, were therefore expected. This could also be an explanation for the lack of significant differences that was observed for the variability outcome measure *Var*. With consistent performances for the participants, in all three groups, the variance should be close to 0 for each participant, i.e., indicating no clear group differences. It should also be noted that the variance curve of three attempts is highly variable due to the low number of replicates, and the corresponding *p*-value function does not show the same smooth behavior as *Mean* and *Max*. Analyses (not presented here) of curves corresponding to the standard deviation and the range gave similar results. The *Max* curve is frequently used when it comes to evaluation of performance (Ryan et al., 2006), and since it actually represents a true hop trial, it is easily interpretable. The *Mean* curve is not as easy to interpret, but in the presence of higher variability between trials it should in theory be more reliable (Markström et al., 2019). Group comparisons are however based on group averages, and hence both these outcome measures provide relevant information. The choice of either *Max* or *Mean* in our data showed disparities, but similar tendencies were still seen for many of the comparisons. This is also reassuring from a clinical interpretation point of view, although there is still a need to determine the best functional test, which will be of even higher clinical value (see below). We compared three different representations, but other options are also described in the literature, e.g., a representative curve based on functional depth (Sangeux and Polak, 2015).

### Choice Regarding Leg Comparison

A choice that has been advocated previously (Hébert-Losier et al., 2015, 2018; Markström et al., 2018), is to compare the injured leg to the non-dominant leg, as it is believed to produce more conservative analysis than if the injured leg was to be compared to the dominant leg. It is also based on the assumption that the dominant leg is more skilled than the non-dominant leg in many tasks. The overall results here confirm this, since we found more differences when comparing to the dominant leg (cf. Table 2). This rationale is however not always supported by the literature when no differences are found between the legs (e.g., Van der Harst et al., 2007; Cug et al., 2016; Mokhtarzadeh et al., 2017). A systematic review by McGrath et al. (2016) demonstrates no significant effect of limb dominance for isokinetic strength of quadriceps and hamstring or for any of the four hop tests included (of which one being the OLVH) in physically active persons/athletes. If there generally were no differences between the dominant and the non-dominant leg of asymptomatic controls, comparisons could be made to either of the legs. This may nevertheless be dependent on the particular task and also requires that the outcome measures are sensitive enough.

Other studies even match leg dominance with regards to injured leg in comparison to controls (Lehmann et al., 2017), and the outcome or choice may even be dependent on specific sports specialization. So this issue still needs further confirmation. To guide clinical evaluation and decision of return to sports, there is certainly a need for better research generated evidence regarding leg comparisons, and provision of valid reference data.

In the current context, it is also relevant to address the issue of how to best determine leg dominance. In the present paper, the dominant leg was defined as the preferred leg for kicking a ball, which is considered an accurate method at least for healthy adults and for bilateral tasks (van Melick et al., 2017). Leg dominance may play a role in relation to ACL injury, but is likely to be context and/or exposure dependent and with no clear-cut interpretations. For example, female soccer players seem more likely to injure their supporting leg, while male players tend to injure their kicking leg (Brophy et al., 2010). Similarly, alpine skiers have been suggested to run a larger risk or injuring their left, often non-dominant leg (Westin et al., 2018).

### Choice of Multiple Joint Comparisons

The strength of the movement analysis based on several joints is the control of the interval-wise error rate by taking into account all statistical tests that are performed. We adjusted for multiple testing, but in a less conservative way than Bonferroni correction. On the other hand, a multi-aspect analysis reduces the power of the test compared to performing analyses of each joint separately; a characteristic shared by all multiple testing procedures (Dudoit and van Der Laan, 2007). In our case, as seen in Table 2, the results from analyses of one joint versus two joints indicated similar group differences, i.e., leading to the same conclusions, implying that the power reduction here did not matter. One should still in general pay attention to the fact that adding several joints to the analysis would on one hand provide more information about data differences, but on the other hand add more variability, possibly rendering the procedure more conservative (Pini et al., 2019). Similarly, including parts of the domain where we do not suspect any differences will also decrease the possibility of detecting significant parts. Here, we analyzed the whole jump from the take-off phase to the landing phase, but if the research question only relates to the take-off or the landing, these should be analyzed separately (as in e.g., Markström et al., 2019).

Several recent reviews (Engelen-van Melick et al., 2013; Dingenen and Gokeler, 2017; Kotsifaki et al., 2020) emphasize the need of considering several different adjacent body segments/joints such as trunk, hip and ankles in the analysis. In the present study we did not observe any major differences depending on choice of the included joints. This topic of how to address joint choices and intertwine the analyses certainly warrants more research with explorations of various movements of different complexity for knee injured persons and asymptomatic controls in order for straightforward interpretations for both researchers and clinicians.

### Strengths and Limitations of the Study

The results presented here provide a thorough investigation of how the discussed methodological choices may impact the results drawn from group comparisons during an OLVH. We focused on the group comparisons, without any additional *post hoc* analyses, in line with the current scope. We only considered one single functional task assessment, which thus can be seen as a study limitation. There were also only three trials, which in general are considered few for motion analysis, but many repetitions are not always possible for maximal performance, neither in research nor in the clinics. Also note that the results/conclusions obtained with our protocol (barefoot and restricted arms) may differ to other studies using different protocols.

Since the OLVH, despite being a whole-body coordination task involving simultaneous control of multiple joints, is rather straightforward to perform–jumping upwards–we would expect to see even more pronounced effects of the methodological choices in evaluation of human performance in other even more complex movement tasks. Indeed, with regards to which specific one leg hop test to choose in clinical evaluation and research, and subsequent to our test protocol, Kotsifaki et al. (2021) recently demonstrated in a comparison of a horizontal one leg hop and a vertical ditto, that the horizontal hop exerts much more demand on the knee, particularly during the landing (65% of the total work). During the vertical hop on the other hand, the hip, knee and ankle equally contributed about 1/3 of the total work. This is also in line with our own recent findings where we demonstrate that one leg side hop landings may be even more decisive for determining knee control (Markström et al., 2021b). Considering this, recommendations for hop tests following knee injury may focus more on one leg hops in the horizontal plane and in particular to the side, while also incorporating adjacent joints, in line with the current paper. A consensus on best rationale for methodological choices would also be beneficial in order to generate evidence in the field.

A strength with the current paper is that curve data are analyzed instead of reducing the observed curves to discrete event-related kinematic variables. Generally, analyses of curve data are superior and more informative for biomechanical interpretation of human motion than discrete event related variables (Ryan et al., 2006). This has been demonstrated by e.g., Richter et al. (2014) who showed that variables/methods that incorporate the sequential nature of the data better explains the jump height in vertical jumping. Further, Godwin et al. (2010) showed that FDA-methods could capture kinematic and kinetic differences not identified using traditional discrete event-related variables.

The implemented testing procedure can be based on different test statistics, and we used standardized test statistics, as many classical multivariate statistical methods (MANOVA, Hotelling’s T, etc.) also do. If we instead would have used test statistics that were not standardized, the result would have changed, since in such cases the result would have been affected by the fact that the outcome measures have different scales. A possibility in such a case would be to standardize data before performing the analysis. One limitation of the statistical method is that it is based on random permutations, here 1,000 permutations. Due to the randomness, the observed *p*-value functions may vary. On the other hand, the Monte Carlo method used to estimate *p*-value functions also allows us to compute its’ standard deviations. The maximum pointwise standard deviation is 0.016 when the *p*-value is 0.5, which decreases to 0.007 when the *p*-value is 0.05.

A common challenge with analyses is the difficulty in how to report and visualize the results, especially in the current case if limited to paper format. Here instead, we present Supplementary Material (see text footnote 1) in different versions in the Shiny App. The possibility for the reader to fully explore the data and the effects of the analysis choices is clearly a strength of our study.

## Conclusion

Our study thus targeted possible impacts on the conclusions depending on the different choice alternatives for the specific outcome measures, leg comparisons and included joints. Normally, the analysis choices of biomechanical research are often implicit in the methods, mainstreamed and/or not always investigated, partly due to the challenges in evaluating human movement control. Despite well-established movement analysis methods, many challenges remain in quantifying, analyzing, and interpreting the often complex data. Our paper, as one of very few scientific contributions to the topic, highlights the consequences of different analysis choices that arise in evaluation of movement performance, in particular when comparing individuals of compromised movement ability with asymptomatic controls. Specifically, our results showed group differences both for *Mean* (a mean curve) and *Max* (a curve corresponding to the highest hop), while less group differences were found for *Var* (a curve describing the variability). Depending on the choice regarding leg comparison, we identified diverse group differences for different joints. Overall, there seemed to be more pronounced disparities when comparing the injured leg to the dominant leg of controls. For a more stringent comparison, we suggest considering to instead compare the injured leg to the non-dominant leg. For our data, the additional value of a multi-joint comparison was however not obvious, since we observed similar results when including multiple joints compared to single joint analysis.

Comparisons based on three-dimensional movement analysis of a vertical hop test are thus influenced by common analysis choices. Specifically, the type of movement curve, leg comparison and included joints all play a role in providing different outcomes among individuals with ACLR, asymptomatic controls and athletes. It is likely that our findings are also relevant for other movement tests and among other populations. The methodology proposed here could be directly applied to other contexts where the observed data are functions. It could be other tasks for similar populations, other populations, or even totally different research areas. Based on our findings, we strongly encourage a wider debate on the strengths and limitations of various analysis choices regarding movement evaluation for pathological and asymptomatic individuals/groups. Further, we strongly recommend well-documented methodological analysis choices with regards to comparisons and representative values of the measures of interests based on clear pre-rationales, and consensus of best-practice in the specific field.

## Data Availability Statement

The data analyzed in this study is subject to the following licenses/restrictions: the dataset presented in this article is not immediately available due to Swedish legislation. Requests to access these datasets should be directed to CH, charlotte.hager@umu.se.

## Ethics Statement

The studies involving human participants were reviewed and approved by the Regional Ethical Review Board in Umeå, Sweden (Dnr. 2015/67-31). Written informed consent was obtained from all participants, including a few underaged of 17 years old, who can take the decision themselves according to the legislation. Their legal guardians received written and oral information.

## Author Contributions

JS, AP, CH, and LS contributed to the idea and wrote the manuscript. AP implemented the multi-aspect IWT method. JS performed the analysis and constructed, together with LS, the graphical illustrations. CH was responsible for the data collection. CH and LS provided the funding. All authors have edited and approved the final version of the manuscript.

## Conflict of Interest

The authors declare that the research was conducted in the absence of any commercial or financial relationships that could be construed as a potential conflict of interest.

## Footnotes

1 https://github.com/Schelin/AnalysisChoicesOLVH

## References

[B1] Abramowicz K. Häger C. K. Pini A. Schelin L. Sjöstedt de Luna S. Vantini S. (2018). Nonparametric inference for functional-on-scalar linear models applied to knee kinematic hop data after injury of the anterior cruciate ligament. *Scand. J. Stat.* 45 1036–1061. 10.1111/sjos.12333

[B2] Abrams G. D. Harris J. D. Gupta A. K. McCormick F. M. Bush-Joseph C. A. Verma N. N. (2014). Functional performance testing after anterior cruciate ligament reconstruction: a systematic review. *Orthop. J. Sports Med.* 2: 2325967113518305. 10.1177/2325967113518305 PMC4555525 26535266

[B3] Banks C. L. Pai M. M. McGuirk T. E. Fregly B. J. Patten C. (2017). Methodological choices in muscle synergy analysis impact differentiation of physiological characteristics following stroke. *Front. Comput. Neurosci.* 11: 78. 10.3389/fncom.2017.00078 28912707 PMC5583217

[B4] Botvinik-Nezer R. Holzmeister F. Camerer C. F. Dreber A. Huber J. Johannesson M. (2020). Variability in the analysis of a single neuroimaging dataset by many teams. *Nature* 582 84–88. 32483374 10.1038/s41586-020-2314-9 PMC7771346

[B5] Brophy R. Silvers H. J. Gonzales T. Mandelbaum B. R. (2010). Gender influences: the role of leg dominance in ACL injury among soccer players. *Br. J. Sports Med.* 44 694–697. 10.1136/bjsm.2008.051243 20542974

[B6] Cug M. Wikstrom E. A. Golshaei B. Kirazci S. (2016). The effects of sex, limb dominance, and soccer participation on knee proprioception and dynamic postural control. *J. Sport Rehabil.* 25 31–39. 10.1123/jsr.2014-0250 26355541

[B7] Dingenen B. Gokeler A. (2017). Optimization of the return-to-sport paradigm after anterior cruciate ligament reconstruction: a critical step back to move forward. *Sports Med.* 47 1487–1500. 10.1007/s40279-017-0674-6 28078610

[B8] Dudoit S. van Der Laan M. J. (2007). *Multiple Testing Procedures With Applications to Genomics.* Berlin: Springer Science & Business Media.

[B9] Engelen-van Melick N. van Cingel R. E. Tijssen M. P. Nijhuis-van der Sanden M. W. (2013). Assessment of functional performance after anterior cruciate ligament reconstruction: a systematic review of measurement procedures. *Knee Surg. Sports Traumatol. Arthrosc.* 21 869–879. 10.1007/s00167-012-2030-6 22581194

[B10] Fleisig G. Chu Y. Weber A. Andrews J. (2009). Variability in baseball pitching biomechanics among various levels of competition. *Sports Biomech.* 8 10–21. 10.1080/14763140802629958 19391491

[B11] Godwin A. Takahara G. Agnew M. Stevenson J. (2010). Functional data analysis as a means of evaluating kinematic and kinetic waveforms. *Theor. Issues Ergon. Sci.* 11 489–503. 10.1080/14639220903023368

[B12] Gokeler A. Welling W. Benjaminse A. Lemmink K. Seil R. Zaffagnini S. (2017). A critical analysis of limb symmetry indices of hop tests in athletes after anterior cruciate ligament reconstruction: a case control study. *Orthop. Traumatol. Surg. Res.* 103 947–951. 10.1016/j.otsr.2017.02.015 28428033

[B13] Greska E. K. Cortes N. Ringleb S. I. Onate J. A. Van Lunen B. L. (2017). Biomechanical differences related to leg dominance were not found during a cutting task. *Scand. J. Med. Sci. Sports* 27 1328–1336. 10.1111/sms.12776 27747935

[B14] Gustavsson A. Neeter C. Thomeé P. Silbernagel K. G. Augustsson J. Thomeé R. (2006). A test battery for evaluating hop performance in patients with an ACL injury and patients who have undergone ACL reconstruction. *Knee Surg. Sports Traumatol. Arthrosc.* 14 778–788. 10.1007/s00167-006-0045-6 16525796

[B15] Hébert-Losier K. Pini A. Vantini S. Strandberg J. Abramowicz K. Schelin L. (2015). One-leg hop kinematics 20 years following anterior cruciate ligament rupture: Data revisited using functional data analysis. *Clin. Biomech.* 30 1153–1161. 10.1016/j.clinbiomech.2015.08.010 26365484

[B16] Hébert-Losier K. Schelin L. Tengman E. Strong A. Häger C. K. (2018). Curve analyses reveal altered knee, hip, and trunk kinematics during drop–jumps long after anterior cruciate ligament rupture. *Knee* 25 226–239. 10.1016/j.knee.2017.12.005 29525548

[B17] Hoffman M. Schrader J. Koceja D. (1999). An investigation of postural control in postoperative anterior cruciate ligament reconstruction patients. *J. Athl. Train.* 34: 130. PMC1322901 16558555

[B18] Kotsifaki A. Korakakis V. Graham-Smith P. Sideris V. Whiteley R. (2021). Vertical and horizontal hop performance: contributions of the hip, knee, and ankle. *Sports Health* 13 128–135. 10.1177/1941738120976363 33560920 PMC8167345

[B19] Kotsifaki A. Korakakis V. Whiteley R. Van Rossom S. Jonkers I. (2020). Measuring only hop distance during single leg hop testing is insufficient to detect deficits in knee function after ACL reconstruction: a systematic review and meta-analysis. *Br. J. Sports Med.* 54 139–153. 10.1136/bjsports-2018-099918 31142471

[B20] Lehmann T. Paschen L. Baumeister J. (2017). Single-leg assessment of postural stability after anterior cruciate ligament injury: a systematic review and meta-analysis. *Sports Med. Open* 3: 32. 10.1186/s40798-017-0100-5 PMC5574832 28853022

[B21] Markström J. L. Grip H. Schelin L. Häger C. K. (2019). Dynamic knee control and movement strategies in athletes and non-athletes in side hops: implications for knee injury. *Scand. J. Med. Sci. Sports* 29 1181–1189. 10.1111/sms.13432 30972848 PMC6850355

[B22] Markström J. L. Schelin L. Häger C. K. (2021a). A novel standardised side hop test reliably evaluates landing mechanics for anterior cruciate ligament reconstructed persons and controls. *Sports Biomech.* 20 213–229. 10.1080/14763141.2018.1538385 30526381

[B23] Markström J. L. Tengman E. Häger C. K. (2018). ACL-reconstructed and ACL-deficient individuals show differentiated trunk, hip, and knee kinematics during vertical hops more than 20 years post-injury. *Knee Surg. Sports Traumatol. Arthrosc.* 26 358–367. 10.1007/s00167-017-4528-4 28337590 PMC5794830

[B24] Markström J. L. Tengman E. Häger C. K. (2021b). Side-hops challenge knee control in the frontal and transversal plane more than hops for distance or height among ACL-reconstructed individuals. *Sports Biomech.* 10.1080/14763141.2020.1869296 [Epub ahead of print]. 33586624

[B25] McGrath T. M. Waddington G. Scarvell J. M. Ball N. B. Creer R. Woods K. (2016). The effect of limb dominance on lower limb functional performance–a systematic review. *J. Sports Sci.* 34 289–302. 10.1080/02640414.2015.1050601 26055387

[B26] Mokhtarzadeh H. Ewing K. Janssen I. Yeow C. H. Brown N. Lee P. V. S. (2017). The effect of leg dominance and landing height on ACL loading among female athletes. *J. Biomech.* 60 181–187. 10.1016/j.jbiomech.2017.06.033 28712544

[B27] Pini A. Vantini S. (2017). Interval-wise testing for functional data. *J. Nonparametr. Stat.* 29 407–424. 10.1080/10485252.2017.1306627

[B28] Pini A. Spreafico L. Vantini S. Vietti A. (2019). Multi-aspect local inference for functional data: analysis of ultrasound tongue profiles. *J. Multivar. Anal.* 170 162–185. 10.1016/j.jmva.2018.11.006

[B29] Preatoni E. Hamill J. Harrison A. J. Hayes K. Van Emmerik R. E. Wilson C. (2013). Movement variability and skills monitoring in sports. *Sports Biomech.* 12 69–92. 10.1080/14763141.2012.738700 23898682

[B30] R Core Team (2019). *R: A Language and Environment For Statistical Computing.* Vienna: R Foundation for Statistical Computing.

[B31] Richter C. Marshall B. Moran K. (2014). Comparison of discrete-point vs. dimensionality-reduction techniques for describing performance-related aspects of maximal vertical jumping. *J. Biomech.* 47 3012–3017. 10.1016/j.jbiomech.2014.07.001 25059895

[B32] Rudolph K. S. Axe M. J. Snyder-Mackler L. (2000). Dynamic stability after ACL injury: who can hop? *Knee Surg. Sports Traumatol. Arthrosc.* 8 262–269. 10.1007/s001670000130 11061293

[B33] Ryan W. Harrison A. Hayes K. (2006). Functional data analysis of knee joint kinematics in the vertical jump. *Sports Biomech.* 5 121–138. 10.1080/14763141.2006.9628228 16521626

[B34] Sangeux M. Polak J. (2015). A simple method to choose the most representative stride and detect outliers. *Gait Posture* 41 726–730. 10.1016/j.gaitpost.2014.12.004 25533050

[B35] Setuain I. Bikandi E. Ruiz F. A. A. Urtasun F. Izquierdo M. (2019). Horizontal jumping biomechanics among elite female handball players with and without anterior cruciate ligament reconstruction: an ISU based study. *BMC Sports Sci. Med. Rehabil.* 11: 30. 10.1186/s13102-019-0142-8 31832206 PMC6859617

[B36] Strandberg J. (2020). *Non-Parametric Methods for Functional Data.* Doctoral dissertation. Umeå: Umeå University.

[B37] Tate J. Suckut T. Wages J. Lyles H. Perrin B. (2017). The associations between hip strength and hip kinematics during a single leg hop in recreational athletes post ACL reconstruction compared to healthy controls. *Int. J. Sports Phys. Ther.* 12: 341. PMC5455184 28593088

[B38] Thomeé R. Neeter C. Gustavsson A. Thomeé P. Augustsson J. Eriksson B. (2012). Variability in leg muscle power and hop performance after anterior cruciate ligament reconstruction. *Knee Surg. Sports Traumatol. Arthrosc.* 20 1143–1151. 10.1007/s00167-012-1912-y 22314862

[B39] Van der Harst J. J. Gokeler A. Hof A. L. (2007). Leg kinematics and kinetics in landing from a single-leg hop for distance. A comparison between dominant and non-dominant leg. *Clin. Biomech.* 22 674–680. 10.1016/j.clinbiomech.2007.02.007 17418922

[B40] van Melick N. Meddeler B. M. Hoogeboom T. J. Nijhuis-van der Sanden M. W. van Cingel R. E. (2017). How to determine leg dominance: the agreement between self-reported and observed performance in healthy adults. *PLoS One* 12: e0189876. 10.1371/journal.pone.0189876 29287067 PMC5747428

[B41] Wang H. Fleischli J. E. Zheng N. (2013). Transtibial versus anteromedial portal technique in single-bundle anterior cruciate ligament reconstruction: outcomes of knee joint kinematics during walking. *Am. J. Sports Med.* 41 1847–1856. 10.1177/0363546513490663 23752955

[B42] Wellsandt E. Failla M. J. Snyder-Mackler L. (2017). Limb symmetry indexes can overestimate knee function after anterior cruciate ligament injury. *J. Orthop. Sports Phys. Ther.* 47 334–338. 10.2519/jospt.2017.7285 28355978 PMC5483854

[B43] Westin M. Harringe M. L. Engström B. Alricsson M. Werner S. (2018). Risk factors for anterior cruciate ligament injury in competitive adolescent alpine skiers. *Orthop. J. Sports Med.* 6: 2325967118766830. 10.1177/2325967118766830 PMC5954346 29780835

