# Supplementary material for: Analysis Choices Impact Movement Evaluation: A Multi-Aspect Inferential Method Applied to Kinematic Curves of Vertical Hops in Knee-Injured and Asymptomatic Persons
Source: Front Bioeng Biotechnol. 2021 May 14;9:645014. doi: 10.3389/fbioe.2021.645014 (PMC8160465; doi:10.3389/fbioe.2021.645014)
Supplement: Supplementary file 1 [file Data_Sheet_1.PDF]

Supplementary Material

1 Details on the statistical method

We describe here the technical details of the extended multi-aspect IWT procedure. For ease of notation, we describe everything for a generic case of observed functional data y, without specifying the outcome measure that was used or the particular joints that were included in the analysis. The same model and analysis were indeed replicated for all outcome measures and all joints.

Let $y_{ij}(t)$ be the functional data (*Mean*, *Max* or *Var*) of the subject i observed at the jump percentage t for the combination of joint and plane j. Here, $i = 1,\dots,n$, $0 \le t \le 1$, and $j = 1,\dots,J$, where J represents the number of jointly tested aspects, and it is equal to three times the total number of joints included in the analysis: if only one joint is included, $J = 3$ since we still have the three planes; if two joints are included $J = 6$ (three planes for each joint), and so on. The model that we used is a functional linear model with scalar covariates:

$$y_{ij}(t) = \beta_{0j}(t) + \beta_{CTRL_j}(t)x_{CTRL_i} + \beta_{ATH_j}(t)x_{ATH_i} + \sum_{l=1}^{L} \beta_{lj}(t)x_{li} + \varepsilon_{ij}(t)\,.$$

In our model, ACL-reconstructed subjects are used as a baseline, and group differences are introduced with the two dummy covariates x_{CTRL_i} and x_{ATH_i}. In particular, $x_{CTRL_i} = 1$ if subject i belongs to group CTRL, $x_{CTRL_i} = 0$ otherwise. Similarly, $x_{ATH_i} = 1$ if subject i belongs to group ATH, $x_{ATH_i} = 0$ otherwise. So, $\beta_{0j}(t)$ is a functional effect common to all subjects in the baseline (i.e, ACLR group), $\beta_{CTRL_j}(t)$ is the functional difference between individuals in the CTRL group and individuals in the ACLR group, $\beta_{ATH_j}(t)$ is the functional difference between individuals in the ATH group and individuals in the ACLR group, x_{lj} are the other scalar covariates taken into account in the analysis (BMI, sex, jump height for the *Max* outcome measure), $\beta_{lj}(t)$ is the corresponding functional effect, and $\varepsilon_{ij}(t)$ are iid functional errors with zero mean.

On the linear model we test if the three groups differ. Specifically, we test for each aspect j, the following hypotheses:

H_{0j}: $\beta_{CTRL_j}(t) = \beta_{ATH_j}(t) = \beta_{CTRL_j}(t) - \beta_{ATH_j}(t) = 0$ against $H_{1j} = H_{0j}^C$

where H_{0j}^C denotes the complementary hypothesis, i.e, at least one of the terms $\beta_{CTRL_j}(t)$, $\beta_{ATH_j}(t)$, or $\beta_{CTRL_j}(t) - \beta_{ATH_j}(t)$ is different from zero.

In our test, the three terms $\beta_{CTRL_j}(t)$, $\beta_{ATH_j}(t)$, and $\beta_{CTRL_j}(t) - \beta_{ATH_j}(t)$ represent the differences between the three groups. If they are all jointly equal to zero at all points t, it means that the groups do not differ during the whole jump.

For the level-1 adjusted p-value function, tests are performed separately for each j; in case of the overall adjusted p-value function, a single test is performed jointly on all j; in case of the level-2 adjusted p-

value function, one test is performed for each j, but a further adjustment is done so that the error rate is controlled jointly for all j.

The test statistic that is used for testing a single j (level-1 p-value function) is based on the sum of the standardized OLS estimators of all groups' differences:

$$T_j^I = \int_I \left(\frac{\hat{\beta}_{CTRL_j}(t)}{se\left(\hat{\beta}_{CTRL_j}(t)\right)}\right)^2 + \left(\frac{\hat{\beta}_{ATH_j}(t)}{se\left(\hat{\beta}_{ATH_j}(t)\right)}\right)^2 + \left(\frac{\hat{\beta}_{CTRL_j}(t) - \hat{\beta}_{ATH_j}(t)}{se\left(\hat{\beta}_{CTRL_j}(t) - \hat{\beta}_{ATH_j}(t)\right)}\right)^2 dt$$

According to IWT, the same test statistic is evaluated on all intervals I of the domain. Then, a nonparametric permutation test is used for computing the p-value, and finally the adjusted level-1 p-value function at point t - that we will further denote as $\tilde{p}_1(t)$ - is computed as the maximum of all p-values of intervals containing the point t.

For the overall test, the same type of IWT-based adjustment is performed, but we use a different test statistic, accounting for all aspects at the same time:

$$T_{overall}^I = \sum_{j=1}^{J} \int_I \left(\frac{\hat{\beta}_{CTRL_j}(t)}{se\left(\hat{\beta}_{CTRL_j}(t)\right)}\right)^2 + \left(\frac{\hat{\beta}_{ATH_j}(t)}{se\left(\hat{\beta}_{ATH_j}(t)\right)}\right)^2 + \left(\frac{\hat{\beta}_{CTRL_j}(t) - \hat{\beta}_{ATH_j}(t)}{se\left(\hat{\beta}_{CTRL_j}(t) - \hat{\beta}_{ATH_j}(t)\right)}\right)^2 dt$$

This leads to the computation of the overall adjusted p-value function $p_{overall}(t)$.

Finally, to compute the level-2 adjusted p-value function $\tilde{\tilde{p}}_j(t)$ we have to perform: a test based on IWT-adjustment on each aspect $j = 1, \dots, J$ separately, on all aspects jointly, as well as on all subsets of aspects (a test for all couples of aspects, a test for all triples of aspects, …). The level-2 adjusted p-value function for aspect *j* at point *t* is then computed as the maximum of all adjusted p-value functions at the same point *t* for all performed tests that include the aspect j (one level-1 test on j, all copules of aspects that include j, all triples of aspects that include j, …).

The three adjusted p-value functions have different properties in terms of error control. In particular, for the level-1 p-value function the control is for each *j* independently. For the overall p-value function, the control is similar, but it is declined for the multivariate test, i.e. we have control if the null hypothesis is true for all *j* simultaneously. Finally, for the level-2 adjusted p-value function, the control is stronger: for any interval of the domain with no differences between groups on any subset of aspects, the probability that such an interval (or part of it) is selected as significant in at least one aspect is also controlled.
